# Understanding the Mental Health Impacts of Climate Change in South Africa: Qualitative Insights From Researchers, Practitioners, Ethicists, and Funders on the Need for a Transdisciplinary Approach

**DOI:** 10.1002/hsr2.73111

**Published:** 2026-09-01

**Authors:** Andrew Tomita, Princess Nyoni, Joyce Mlay, Jessica Garlick, Rosebub Gugulethu Gcaba, Thobile Maphumulo, Helga Chauke, Richard Lessells, Admire Nyamwanza

**Affiliations:** ^1^ Centre for Research in Health Systems, School of Medicine University of KwaZulu‐Natal Durban South Africa; ^2^ KwaZulu‐Natal Research and Innovation Sequencing Platform (KRISP), School of Medicine University of KwaZulu‐Natal Durban South Africa; ^3^ School of Medicine University of KwaZulu‐Natal Durban South Africa; ^4^ Department of Community Health Nursing Kairuki University Dar es Salaam Tanzania; ^5^ Department of Public Health Medicine, School of Medicine University of KwaZulu‐Natal Durban South Africa; ^6^ Health Economics and HIV and AIDS Research Division (HEARD) University of KwaZulu‐Natal Durban South Africa; ^7^ Discipline of Physiology, School of Medicine University of KwaZulu‐Natal Durban South Africa; ^8^ Institute of Natural Resources Pietermaritzburg South Africa; ^9^ Institute for Global Health and Development Queen Margaret University Edinburgh UK

**Keywords:** climate change, mental health, Sub‐Saharan Africa

## Abstract

**Background:**

Against a backdrop of entrenched structural inequalities, poverty, and limited healthcare resources, climate change represents a major public health challenge in South Africa, with significant yet under‐recognized consequences for mental health. Expert perspectives on these impacts remain limited, highlighting a critical gap in informing transdisciplinary responses.

**Objectives:**

To explore stakeholder perspectives (researchers/academics, research ethics committee members, employees of organization that funds climate or health research) on transdisciplinary responses to the mental health impacts of climate change in South Africa.

**Methods:**

This exploratory qualitative study examined expert perspectives on the mental health impacts of climate change. Twelve purposively sampled stakeholders participated in in‐depth semi‐structured interviews. Data collection continued until data saturation was reached (*n* = 12). Data were analyzed using an inductive thematic approach.

**Findings:**

Three overarching themes were identified. First, direct mental health impacts included anxiety, depression, trauma, and exacerbation of pre‐existing conditions, with links to severe mental illness, including climate‐related delusional symptoms and increased substance use. Second, indirect pathways included socioeconomic vulnerability, community resilience, and the dual role of media in raising awareness and contributing to psychological distress. Third, participants emphasized the need for strengthened transdisciplinary approaches.

**Conclusions:**

Addressing mental health impacts of climate change requires integrated, contextually relevant, transdisciplinary interventions to strengthen resilience among vulnerable communities.

## 1 Introduction

Climate change has emerged as one of the most pressing threats to global public health with consequences for mental health and psychological well‐being [1]. Exposure to extreme weather events, such as floods, droughts, and heatwaves, can directly affect mental health through acute stress responses [2, 3], psychological trauma associated with loss [4], displacement [5], perceived threats to life [6], and physiological stress that may disrupt brain function and mood regulation [7]. These acute impacts do not occur in isolation, as climate‐related stressors may also exacerbate pre‐existing mental health conditions through mechanisms such as cumulative allostatic load (the physiological impact of repeated stress) [7], the stress‐diathesis model [8] whereby environmental stressors trigger vulnerability in those already predisposed to mental illness, and the gradual depletion of individual and community coping resources.

In South Africa, as a hotspot with one of the highest rates of inequality in the world [9], emerging empirical evidence has reported increased rates of trauma and post‐traumatic stress disorder (PTSD) [10], anxiety [10], depression [11], implications for severe mental illness [12], and substance use and abuse [13] following climate‐related events, particularly flooding and periods of extreme heat. These impacts are concentrated among the most vulnerable population groups, including individuals living in poverty [14, 15], those experiencing homelessness [14], and residents of informal settlements [14]. These groups face increased exposure to environmental hazards [15] alongside limited access to healthcare [14, 15], infrastructure [15], and social support systems [14, 15], further compounding their vulnerability.

South Africa is highly vulnerable to climate change, with devastating climate‐related events such as the 2022 KwaZulu‐Natal floods and the increasing frequency of drought and heatwaves [15] that worsen an existing mental health burden [12]. However, research at this intersection of climate change and mental health in a South African context remains limited and siloed [16]. Additionally, the perspectives of stakeholders who shape research priorities, allocate resources, and govern responses to climate related mental health challenges remain largely absent from the literature. While lived experience is essential for understanding climate‐related mental health impacts, expert stakeholders across research, research ethics, and funding sectors are uniquely positioned to identify systemic gaps, inform priority setting, and support integrated responses that are evidence‐based and contextually grounded. Consequently, this study addresses the following central research question: How do key health and climate stakeholders perceive the mental health impacts of climate change and the requirements for a transdisciplinary response framework in South Africa? Specifically, this study aims to: 1) explore expert stakeholder perspectives on the direct and indirect mental health impacts of climate change in South Africa, and 2) identify systemic levers and multisectoral strategies required to build a resilient, transdisciplinary response.

## 2 Methods

### 2.1 Study Design and Setting

This study was a qualitative study conducted in South Africa using virtual key informant interviews with experts working on climate change and/or mental health research in South Africa.

### 2.2 Target Population Participants

Three types of expert stakeholders in South Africa were approached.

i. researchers/academics working in climate change and/or health;
ii. research ethics committee members (REC) with experience in reviewing climate‐related or health research; and
iii. employees of organization that funds climate change and/or health research.

### 2.3 Sampling Strategy

Participants were identified based on purposive sampling through publicly available sources, including institutional and organizational websites and journal publications, from which names and contact details of relevant experts were obtained. Identified individuals based on the above‐mentioned three criteria were then invited to participate directly.

### 2.4 Data Collection

Data were collected through audio‐recorded in‐depth, semi‐structured interviews conducted via virtual online meeting platforms. Interviews explored participants' perspectives on the relationship between climate change and mental health, particularly the observed impacts of climate change on mental health. Each interview lasted approximately 30 min to 1 h. All interviews were conducted in English by a trained research assistant and were audio‐recorded with the participant's permission, guided by a semi‐structured interview guide. Basic demographic information was also collected from participants. Data collection continued until data saturation was reached at 12 participants, at which point no new themes or insights emerging from the interviews.

### 2.5 Data Analysis

Demographic data were analyzed descriptively while qualitative data were analyzed using an inductive thematic analysis approach to identify patterns and themes related to climate change and mental health. All interviews were transcribed verbatim prior to analysis. NVivo (version 15) software was used to manage and organize the data. Two analysts independently reviewed the transcripts and cross‐checked them against the audio recordings to ensure accuracy and correct any errors. The same two researchers independently coded the transcripts using an inductive approach, allowing themes to emerge directly from the data. Initial codes were generated from the raw data by identifying meaningful units of information within participant responses. Related codes were then grouped according to shared meaning, from which subthemes and overarching themes were derived. For example, quotes describing psychological distress following floods were coded as “disaster related psychological distress,” which together with codes such as “trauma from flood experience” and “increasing worry due to uncertainty of climate change effects” generated the subtheme “causes of anxiety and depression” under the overarching theme of direct effects of climate change on mental health. The full coding framework, including exemplary quotes, codes, code meanings, subthemes, and themes, is presented in Table 1. Coding discrepancies were discussed and resolved to ensure consistency and credibility of the analysis, as described further under Researcher Reflexivity and Positionality.

**Table 1:** Thematic analysis table.

Exemplary quotes	Codes	Meaning of codes	Sub‐theme	Theme
*“There are issues associated with anxiety and depression, both from the impact of a disaster directly.”*	Disaster‐related psychological distress	Psychological suffering caused directly by disasters	Causes of anxiety and depression	Direct effects of climate change on mental health.
*“After the flooding that happened in KZN, there have been more people with reported anxiety, but also depression*.	Trauma from floods experience	Emotional distress following flooding events.		
*“I'd see like the farmers and people with livestock and all that, they start worrying a lot, like having a lot of anxiety, you know, whether it's going to rain or whether it's going to be hot or when it's hot. Too hot like that distresses their livestock. So yeah, I think it does impact people's mental health.”*	Increasing worry due to the uncertainty of the effects of climate change	Ongoing worry due to unpredictable climate conditions.		
*“I mean, if someone has schizophrenia, for example, then the underlying condition is going to be exacerbated by the stress because of climate change”*.	Worsening mental health condition	Climate stress worsens existing mental illness	Exacerbation of pre‐existing mental health conditions	
*“It's not just the heat or the cold that is affecting them; it is also the psychosocial stress associated with climate change. So, I think the mentally ill are more vulnerable now, you know, not coping with those things. They may have less support.”*	Increasing vulnerability of mentally ill patients	Increased susceptibility due to low support and coping capacity		
*“Can have delusions about climate change. So, the delusions that, you know, somebody is purposely creating climate change, controlling the world, because the delusions are based on what they are reading about in the newspapers and watching television”*.	Emerging psychotic symptoms (false beliefs)	Climate information influencing psychotic beliefs	Climate change‐related delusional symptoms	
*“The heat‐related mental health effects increase and substance abuse.”*	Maladaptive coping	Coping responses to climate stress with negative behavior.	Increased use of Substance abuse.	
*“So, the people in [redacted] have little; they have lost everything, and they do not have the resources or the networking to support the level that something in California would be. Do you see the difference? And the resilience is also different.”*	Lack of resources	Lack of social and material support after disasters	Contextual differences in mental health impacts	Indirect effects of climate change on mental health
*“Climate change indirectly increases stress through food insecurity and unemployment.”*	Poverty/food insecurity and unemployment	Socio‐economic stress		
*I think that with social media and people being more willing to talk about mental health issues, it's becoming a more common topic of discussion, which is good.”*	Mental health awareness	Social media improves mental health awareness.	Enhanced social media discourse on climate change and mental health	
*“You know, I think we must not have this alarmist approach that, oh, we are all going to melt away, or we're all going to freeze or whatever”*	Fear and worry about climate change‐related effects.	Fear and worry caused by media coverage of climate change.		
*“For us to stop working in silos, we need to create teams that include people from different aspects*.”	Working in silos, multidisciplinary teams, and collaboration across fields	Research should move away from isolated disciplinary work toward integrated teamwork across fields	Interdisciplinary approach	A collaborative intervention approach to mental health research and climate change
*“Health research must also be conducted with people from public health, environment, and social science… stakeholders from the Department of Health that will align policy with research outcome.”*	Stakeholder involvement; policy alignment; collaborative research	Research should include stakeholders to ensure alignment between evidence generation and policy development	Multi‐stakeholder engagement for policy and practice	

### 2.6 Ethical Consideration

Written informed consent was obtained from all participants prior to participation. A blank consent form was distributed via email by our research team, and participants provided consent by returning a signed electronic copy. Individuals who did not provide consent were excluded. The study was approved by the Biomedical Research Ethics Committee at University of KwaZulu‐Natal (BREC/00007907/2024).

### 2.7 Study Rigor

To ensure the rigor of the study, four criteria of trustworthiness including credibility, dependability, confirmability, and transferability [17] were applied.

Credibility was enhanced through the use of a semi‐structured interview guide, which allowed for in‐depth exploration of participants' perspectives, and by purposively including experts with diverse backgrounds in climate change and mental health research, enabling data source triangulation across varied perspectives. Independent coding by two researchers, followed by discussions to resolve discrepancies, further strengthened the credibility of the findings. In addition, member checking was conducted during interviews by restating participants' responses to confirm accurate representation of their views, and peer debriefing with colleagues was undertaken to challenge assumptions and enhance the robustness of interpretations.

Dependability was ensured by maintaining a clear and consistent research process, including detailed documentation of data collection and analysis procedures. The use of verbatim transcription and a systematic coding process supported consistency and transparency in the analytical approach.

Confirmability was achieved by grounding findings in the data, supported by verbatim transcripts and an inductive analytical approach. Representative participant quotes are presented to illustrate the link between the data and the study's interpretations. Ongoing discussions between researchers during coding helped minimize individual bias and ensured that interpretations remained data‐driven.

Transferability was addressed by providing a clear description of the study context, participant characteristics, and research procedures, enabling readers to assess the applicability of the findings to other settings. Findings were also linked to existing literature to enhance contextual relevance and support broader interpretation.

### 2.8 Researcher Reflexivity and Positionality

The research team had prior experience in mental health research and remained aware that their prior knowledge could influence the research process. To minimize the imposition of pre‐existing interpretations on the findings, data collection was conducted by trained research assistants with no prior experience in climate change and mental health research, who were also instructed not to impose their values on participants' responses. During data analysis, independent coding by multiple researchers, followed by structured discussions to compare interpretations and resolve discrepancies through consensus, served as a deliberate reflexive strategy to ensure that the emerging findings were grounded in participants' own perspectives rather than shaped by the research team's prior assumptions about climate change and mental health.

### 2.9 Findings

A total of 12 experts participated in the study, the majority of whom were female (*n* = 9). Participants represented a broad range of professional experience and disciplinary backgrounds. Most held PhD qualifications (*n* = 10), with additional representation from Master's and Bachelor's degree holders. The majority were researchers/academics, alongside employees of the organization that funds climate change and/or health research; in this case, the latter were all government employees providing diverse perspectives on climate change and mental health. The subject expertise included climate change, ethics, ecology, environmental science, psychology, mental health/psychiatry, epidemiology/biostatistics and public/population health. The demographic characteristics of the study participants are presented in Table 2.

**Table 2:** Sociodemographic Characteristics of study participants.

Sex:	Female	9 (75%)
	Male	3 (25%)
Age group:	30–40	3 (25%)
	40–50	5 (42%)
	Above 50	4 (33%)
Highest level of education:	PhD	10 (83%)
	Master's degree	1 (8%)
	Bachelor's degree	1 (8%)
Professional background	Researchers/Academics	7 (58.33%)
	Research ethics committee member	2 (16.67%)
	Employees of organization that funds climate change and/or health research	3 (25.00%)

*Note:* It is possible for a participant to fall under multiple categories. All employees of organizations that fund climate change and/or health research were from government.

Following the presentation of participants' characteristics in Table 1, the findings from the qualitative analysis are presented below. Participants viewed climate change as a complex and multifaceted issue with implications for mental health that extend beyond environmental exposures alone. Their accounts highlighted both direct and indirect pathways through which climate‐related events and disruptions may affect psychological well‐being, while also emphasizing the need for coordinated responses across disciplines and sectors. Three overarching themes emerged from the analysis: (1) direct effects of climate change on mental health, (2) indirect effects of climate change on mental health and (3) collaborative approaches to climate change and mental health research and intervention.

Theme 1 **Direct effects of climate change on mental health**

Participants identified a range of direct mental health impacts associated with climate change. These included causes of anxiety and depression, worsening of existing mental health conditions, climate change–related delusional symptoms and increased vulnerability to substance use.

***Sub‐theme 1: Causes of anxiety and depression***

Participants explained that climate‐related disasters, particularly floods, have a direct and immediate impact on mental health. They described how such events can lead to psychological consequences, including trauma, anxiety, and depression. These experiences highlight the significant emotional distress that often follows direct exposure to climate disasters, placing a substantial burden on affected individuals' psychological well‐being.

There are issues associated with anxiety and depression, both from the impact of a disaster directly.

(P8, male, researcher/academic)

A participant repeatedly linked flooding events with psychological distress, particularly anxiety, depression, and trauma.

Floods that directly contribute to mental health consequences such as trauma, anxiety, and depression.

(P10, female, an employee of organization that funds climate change and/or health research)

One participant further reflected on the KwaZulu‐Natal floods, noting an increase in reported mental health challenges following the disaster.

After the flooding that happened in KZN, there have been more people with reported anxiety, but also depression.

(P5, female, researcher/academic)

Furthermore, participants noted that the increasing occurrence and intensity of events such as hurricanes, wildfires, droughts, and floods are closely associated with experiences of trauma, anxiety, and depression. Similarly, another participant emphasized the severity of mental health challenges, noting the effects of heat.

The increasing occurrence and severity of climate‐related phenomena such as hurricanes, wildfires, droughts, and floods directly contribute to mental health consequences such as trauma, anxiety, and depression.

(P11, male, researcher/academic)

The consequences on mental health are really high. Heat also aggravates people.

(P3, female, researcher/academic)

In addition, participants highlighted that climate change has negatively affected mental health, noting that people are becoming more anxious and depressed due to a strong sense of helplessness and lack of control over the situation. Another participant further emphasized that this impact is particularly evident among farmers and livestock keepers, who experience anxiety due to uncertainty about weather conditions, including worries about whether it will rain or become excessively hot, and the potential effects of extreme heat on livestock.

I think more people are anxious, more people are depressed, because I think the sense is that they can't do anything about it, that it's completely out of their control.

(P2, female, research ethics committee member)

I'd see like the farmers and people with livestock and all that, they start worrying a lot, like having a lot of anxiety, you know, whether it's going to rain or whether it's going to be hot or when it's hot. Too hot like that distresses their livestock. So yeah, I think it does impact people's mental health.

(P4, female, researcher/academic)

Participants further explained that the mental health burden is often greater among vulnerable populations, who are more likely to experience the compounded impacts of climate‐related events. They highlighted that repeated exposure to such events can lead to persistent anxiety and chronic stress, particularly due to the fear of recurrence. In addition, participants noted that experiences such as the loss of family members during these events contribute to trauma and post‐traumatic stress, further intensifying mental health challenges.

So, if we are having extreme climate changes that affect our Physiology and, in turn, affect our mental health.

(P1, male, researcher/academic)

Anxiety and the chronic stress that some of our patients come in with, because they then have the fear of recurrent occurrences [of climate events].

(P9, male, an employee of organization that funds climate change and/or health research)

Participants further noted that the psychological consequences of climate‐related events may be intensified by experiences of loss and by the heightened vulnerability of populations with limited resources and support systems.

Losing family members in those events as well. Then they also come in with trauma and post‐traumatic stress disorders.

(P12, female, an employee of organization that funds climate change and/or health research)

So, I think obviously, I mean, especially the extreme weather will be causing big problems for the homeless or vulnerable, they may have more anxiety, they may have more psychosocial thoughts.

(P7, female, research ethics committee member)

Contextually, these insights reveal that eco‐anxiety and climate distress in South Africa are not abstract concepts. Instead, they are intimately linked to immediate existential threats. Collectively, these accounts suggest that participants perceived climate change as contributing to psychological distress through both acute and chronic pathways. Acute climate‐related events, such as floods and extreme weather were viewed as triggering immediate emotional responses including trauma, anxiety and depression, while ongoing uncertainty regarding future climate conditions contributed to persistent stress and feelings of helplessness. Participants further emphasized that these impacts may be unevenly distributed, with vulnerable populations including those with limited resources and climate‐sensitive livelihoods perceived to face a greater psychological burden. The findings also suggest that repeated exposure to climate‐related events may compound mental health challenges through ongoing fears of recurrence, loss and disruption.

***Sub‐theme 2: Exacerbation of Pre‐existing Mental Health Conditions***

Two participants emphasized that psychosocial stress arising from climate‐related disruptions plays a critical role in worsening mental health outcomes. Individuals living with mental illness were perceived as particularly vulnerable, as climate change introduces additional stressors that may exceed their coping capacity. They also highlighted that limited social and healthcare support further heightens the risk of deterioration in mental well‐being. Overall, climate change was viewed as exacerbating pre‐existing mental health conditions and intensifying psychological distress, including individuals diagnosed with schizophrenia.

I mean, if someone has schizophrenia, for example, then the underlying condition is going to be exacerbated by the stress because of climate change.

(P7, female, research ethics committee member)

Also, heat stress‐related mental health effects that you will see in our clients are being irritable and sleep disturbances, and also, worsening of their existing mental illness.

(P12, female, an employee of organization that funds climate change and/or health research)

Expanding on this, P7 highlighted that the vulnerability of individuals with pre‐existing mental health conditions may stem not only from environmental exposures, but also from the psychosocial stress associated with climate‐related disruptions. The participant further suggested that limited social support may make it more difficult for individuals to cope with these challenges.

It's not just the heat or the cold that is affecting them; it is also the psychosocial stress associated with climate change. So, I think the mentally ill are more vulnerable now, you know, not coping with those things. They may have less support.

(P7, female, research ethics committee member)

Participants perceived climate change as interacting with pre‐existing mental health conditions in ways that may increase vulnerability to adverse outcomes. Rather than affecting all individuals equally, climate‐related stressors were viewed as placing a disproportionate burden on those already living with mental illness particularly where social and healthcare support systems are limited. Participants highlighted both environmental exposures, such as heat and broader psychosocial stressors as potential pathways through which existing symptoms may worsen.

***Sub‐theme 3: Climate Change–Related Delusional Symptom***

One participant highlighted that climate change may influence not only emotional distress but also the content of delusional experiences among individuals with psychotic disorders. They noted that widespread media coverage and public discussions about climate change can become incorporated into delusional thinking, leading some individuals with psychotic disorders to develop false beliefs that climate change is being intentionally created by powerful forces. In this context, powerful forces refers to the beliefs arising because of psychotic delusions, rather than the real influence that governments or other authorities may have on climate change through policy, legislation, or environmental decision‐making. This may shape psychotic symptoms, reflecting how broader environmental crises can affect the psychological experiences of those living with severe mental illness.

Can have delusions about climate change. So, the delusions that, you know, somebody is purposely creating climate change, controlling the world, because the delusions are based on what they are reading about in the newspapers and watching television.

(P7, female, research ethics committee member)

Although raised by a single participant, this observation suggests that climate change may influence not only emotional well‐being but also the content of psychotic experiences among some individuals with severe mental illness. The participant described how widespread media coverage and public discourse surrounding climate change may become incorporated into existing delusional beliefs. Rather than suggesting a direct relationship between climate change and psychosis, this perspective highlights how prominent societal concerns may shape the themes and content of delusional thinking.

***Subtheme 4: Increased use of substance abuse***

One participant noted that climate change–related stressors, particularly extreme heat, may increase vulnerability to substance use among individuals experiencing mental health challenges. One participant linked heat‐related psychological distress to substance abuse, suggesting that worsening environmental conditions can intensify symptoms and overwhelm coping capacity. This highlights how climate change may contribute indirectly to mental health risks through increased reliance on harmful coping behaviors.

The heat‐related mental health effects increase and substance abuse [also increases].

(P12, female, an employee of organization that funds climate change and/or health research)

This account suggests that climate‐related stressors may affect mental health not only directly but also through their influence on coping behaviours. The participant perceived extreme heat as contributing to psychological distress that could increase susceptibility to substance use among vulnerable individuals. While raised by a single participant, this observation points to a potential pathway linking climate‐related stress and adverse behavioural health outcomes.

Theme 2 **Indirect effects of climate change on mental health**

Participants described how the mental health impacts of climate change are shaped by broader social, economic and contextual factors. They highlighted differences in vulnerability and resilience across communities, while also reflecting on the growing visibility of climate change and mental health within social media and public discourse. Two sub‐themes emerged from the analysis: (1) contextual differences in mental health impacts and (2) enhanced social media discourse on climate change and mental health.

*Sub‐theme 1: Contextual Differences in Mental Health Impacts*

Participants emphasized that the emotional and psychological effects of climate‐related events vary across communities, depending on location and available resources. They explained that the impact of a disaster is not determined solely by the event itself, but also by its socioeconomic consequences. In communities with limited resources, such as informal settlements, individuals often face greater distress because they lack the financial, social, and institutional support needed to cope. This lack of support can exacerbate anxiety, depression, and trauma, making recovery more difficult and prolonged. In contrast, communities with greater access to resources and support networks are better able to manage the psychological burden of similar events. Overall, these accounts highlight that contextual differences strongly shape both the severity of mental health impacts and the capacity for recovery following climate‐related disasters.

So, the people in [redacted] have little; they have lost everything, and they do not have the resources or the networking to support the level that something in California would be. Do you see the difference? And the resilience is also different.

(P10, female, an employee of organization that funds climate change and/or health research)

Because, you know, they are already so poor and they're malnourished, and they don't have resources and health care facilities.

(P10, female, an employee of organization that funds climate change and/or health research)

Beyond the immediate impacts of climate‐related events, participants also highlighted how existing socioeconomic challenges can further compound psychological distress. One participant noted that climate‐related disruptions may increase stress through their effects on livelihoods and access to basic resources:

Climate change indirectly increases stress through food insecurity and unemployment.

(P7, female, research ethics committee member)

Participants further noted that climate‐related vulnerabilities are not experienced equally across populations. One participant highlighted that women and children may be particularly vulnerable to the broader impacts of climate change due to existing social and economic inequalities.

You know, facing inequality. You've got marginalized groups such as women and children, especially children under five. So those are the groups that are mostly affected.

(P6, female, academic/researcher)

Expanding on this, another participant emphasized that climate‐related disruptions may affect women and children through their impact on household food security and access to adequate nutrition.

And, so when we add all of these together, so then we should talk about who becomes the most vulnerable in the household, especially it's going to be women and children. If we are talking about women and children, what happens to their access to, you know, food that's rich in micronutrients.

(P11, male, researcher/academic)

Also, participants indicated that the mental health impacts of climate change disasters vary according to community resilience. In communities that have experienced repeated climate‐related events, resilience has often been built through hardship; however, this accumulated adversity directly affects how individuals cope with stress.

I think Global South, the Asian countries, and in Africa, there is a resilience that we undermine and do not recognize because we benchmark against European and American people's values and standards.

(P10, female, an employee of organization that funds climate change and/or health research)

Is that person's psychological, you know, makeup in terms of personality factors, in terms of their defenses, in terms of their coping skills, their resilience.

(P10, female, an employee of organization that funds climate change and/or health research)

Can live in poverty, but not everyone will become depressed.

(P7, female, research ethics committee member)

These insights emphasize that vulnerability and resilience do not exist in a vacuum, but are products of historical, structural inequality. When participants contrast South African townships with affluent regions like California, they underscore that the severity of mental health trauma is fundamentally mediated by infrastructure. Resilience in the Global South often manifests as a forced adaptation to chronic hardship rather than an optimized state of well‐being. Ultimately, this tells us that mental health interventions cannot succeed through clinical psychotherapy alone but must actively address the structural determinants of health such as food security, housing integrity and equitable resource allocation.

***Sub‐theme 2: Enhanced social media discourse on climate change and mental health***

One participant noted that social media had become a significant space for discussing climate change and its psychological consequences. The participant cautioned that some messages shared on these platforms may create fear and worry. This highlights the need for careful communication when discussing climate change and mental health on social media.

You know, I think we must not have this alarmist approach that, oh, we are all going to melt away, or we're all going to freeze or whatever.

(P10, female, an employee of organization that funds climate change and/or health research)

In addition, two participants further described how the growing discussion on social media has contributed to a shift, making mental health concerns linked to climate change more visible and increasingly recognized in ongoing discussions. They noted that such discussions can also raise awareness of the mental health effects of climate change.

I think that with social media and people being more willing to talk about mental health issues, it's becoming a more common topic of discussion, which is good.

(P10, female, an employee of organization that funds climate change and/or health research)

From a mental health perspective, there is currently quite a lot of awareness, in terms of talks in communities.

(P5, female, researcher/academic)

Theme 3 **A collaborative intervention approach to mental health research and climate change**

Participants emphasized that the mental health impacts of climate change cannot be adequately addressed through isolated disciplinary or sectoral efforts. Instead, they advocated for collaborative approaches that bring together diverse forms of expertise, knowledge systems, and stakeholder perspectives to better understand and respond to climate‐related mental health challenges. Two sub‐themes emerged from the analysis: (1) interdisciplinary approaches and (2) multi‐stakeholder engagement for policy and practice.

***Sub‐theme 1: Interdisciplinary approach***

Participants emphasized the importance of bringing together experts from diverse disciplines, including public health, environmental sciences, social sciences, and mental health, to better understand and address the complex relationship between climate change and mental health. They highlighted that climate change and health are interconnected systems that require collaborative research approaches rather than discipline‐specific efforts.

For us to stop working in silos, we need to create teams that include people from different aspects.

(P7, female, research ethics committee member)

The people who can provide you with the information. They do not come from a single discipline or field, nor from a particular government department or practitioner's field, so it is essential to have this transdisciplinary process.

(P6, female, researcher/academic)

Building on the importance of collaboration across disciplines and sectors, one participant further argued that addressing climate‐related mental health challenges requires a systems approach that considers the interconnected nature of environmental, social, and health processes.

The area of climate and health requires a systems approach. You have to think of many different dimensions simultaneously, and you have to understand that if something happens in part of the system, the consequences will be in other parts of the system. It also involves transdisciplinary research, which means working across multiple disciplines.

(P8, male, researcher/academic).

***Sub‐theme 2: Multi‐stakeholder Engagement for Policy and Practice***

Participants stressed the importance of involving a broad range of stakeholders, including government departments, practitioners, civil society organizations, and communities, throughout the research process. Such engagement was viewed as essential for ensuring that research findings are translated into policies, interventions, and practical responses that address the mental health consequences of climate change.

So, health research must also be conducted with people from public health, environment, and social science, and from, you know, stakeholders from the Department of Health that will align policy with whatever the research outcome is.

(P7, female, research ethics committee member)

You have to work with government stakeholders, with practitioners, and civil society.

(P8, male, researcher/academic).

## 3 Discussion

This study advances understanding of the mental health impacts of climate change in South Africa by drawing on the perspectives of a uniquely diverse expert group, one that notably extends beyond the conventional academic and clinical voices to include research funders and ethics committee members, whose insights illuminate the systemic and governance dimensions of this challenge often absent from the literature. The findings suggest that climate change disrupts mental health through direct and indirect pathways, with disproportionate consequences for already vulnerable populations. Critically, while much of the existing literature has concentrated on common mental health outcomes such as anxiety, depression, and stress, this study navigated relatively neglected terrain: the impact of climate change on severe mental illness, including its potential to shape the content of psychotic and delusional symptoms and its role in driving substance use, both of which remain significantly understudied in the South African context. The findings further support the belief that these psychological consequences cannot be understood in isolation from the broader social and structural conditions in which they unfold, including poverty, food insecurity, constrained healthcare access, and marked community‐level differences in resilience, all of which mediate both the severity of distress and the capacity for recovery. Together, these findings underscore that climate‐related mental health impacts in South Africa are not simply clinical phenomena but are fundamentally shaped by deep structural inequalities, a reality that demands integrated, contextually grounded responses. Therefore, our findings indicate that climate‐related mental health impacts are biopsychosocial manifestations of structural inequality.

In terms of the direct effects of climate change on mental health, participants described how climate‐related disasters, particularly floods and extreme heat, contribute to a range of psychological consequences including trauma, PTSD, anxiety, and depression, a finding that resonates with existing literature linking environmental disasters to psychological deterioration [5, 18]. This is consistent with disaster mental health literature, mostly from the Global North, showing that acute environmental events are associated with anxiety, depression, PTSD, resource loss and an increased need for mental health services, including hospital admissions (Goldman & Galea, 2014; [6, 13]). However, the present study adds a South African perspective by showing how participants understood these mental health effects as occurring within conditions of poverty, repeated climate‐related stress, pre‐existing mental illness, and limited access to healthcare. Beyond acute exposure, participants highlighted that anxiety often persists due to fear of future climate events, indicating that psychological impacts extend well beyond the immediate disaster period. Participants also highlighted that from their experience, individuals with pre‐existing mental illness, particularly those with schizophrenia, were especially vulnerable, as climate‐related stressors were seen to compound existing symptom burden and exceed coping capacity in contexts where healthcare resources in South Africa are limited. These experiences and perceptions are consistent with previous research indicating that heat exposure is a clinically relevant climate‐related stressor for those living with schizophrenia. In a case‐crossover study, Tupinier Martin et al. [19] found that hot temperatures were associated with increased hospital admissions for psychosis among adults diagnosed with schizophrenia. A particularly novel finding was the discussion of climate‐related delusional symptoms among individuals with psychotic disorders, whereby climate change coverage encountered through media and public discourse appeared to become incorporated into the content of their delusional belief systems. This is consistent with the well‐established clinical concept of the cultural shaping of delusions, which holds that the topics that appear in the delusions of people with psychotic disorders are shaped by what is prominent in their surrounding world, including media coverage and public discourse [20, 21]. While reported by a single participant and warranting cautious interpretation, this finding has meaningful clinical implications and calls for further investigation. Finally, extreme heat and other environmental stressors were associated with increased reporting of substance use as a maladaptive coping strategy, illustrating how climate change can simultaneously trigger new mental health challenges and aggravate existing disorders. These observations support growing evidence that climate change acts not only as a population‐level stressor but also shapes clinical presentations and mental health trajectories, highlighting potential implications for community well‐being and mental health care.

With respect to the indirect effects of climate change on mental health, our findings highlight that the psychological consequences of climate change are powerfully shaped by context. Participants emphasized that socioeconomic conditions, including poverty, food insecurity, and limited healthcare access, add to mental health vulnerability in under‐resourced communities, with specific reference to urban townships in South Africa, where residents face compounding climate‐related risks including severe flooding, shack fires, inadequate sanitation, and environmental pollution, all of which are experienced against a backdrop of persistent poverty and limited institutional support [22]. This contextual emphasis is supported by African and South African evidence, with Deglon et al. [16] reporting that the African evidence repeatedly identifies vulnerability among those living in poverty, farmers, pastoralists, women and children. South African studies similarly show that flood and disaster‐related mental health risks are influenced by food insecurity, infrastructure loss, prior trauma, low income and cumulative community disaster exposure [10, 11]. Therefore, these findings are consistent with established literature on the social determinants of mental health [23, 24], while adding context‐specific evidence on how structural inequality influences climate‐related mental health vulnerability in South Africa. Communities in the Global South, including those in South Africa, were perceived by participants to have developed meaningful forms of resilience through lived experience of hardship, yet the distinctive resilience that has emerged within these communities through generations of adversity often goes unrecognized because it does not conform to Western standards of coping and recovery [24]. Participants noted, however, that while individuals may demonstrate remarkable resilience even in the context of poverty, the presence of structural inequalities such as food insecurity and unemployment means that not everyone is equally protected from the psychological consequences of climate‐related events.

One group identified by participants as potentially facing heightened vulnerability was women and children. Participants highlighted women and children as particularly vulnerable to the broader consequences of climate change, including food insecurity, nutritional deprivation and other resource‐related challenges. Although gender did not emerge as a major focus of the interviews, this observation aligns with evidence suggesting that climate change often disproportionately affects women due to existing social and economic inequalities, caregiving responsibilities and differential access to resources [25]. Compared with accounts that focus mainly on differences in climate concern or worry, participants in this study framed women's vulnerability through more material and relational pathways, including food insecurity. This aligns with African and global literature showing that climate vulnerability is influenced by gendered roles, agency and resource access with women having higher levels of climate‐related concern and worry than men [25, 26, 27]. Future research in South Africa should further explore how gender shapes experiences of climate‐related mental health impacts, including potential differences in vulnerability, coping strategies and help‐seeking behaviours.

A further indirect pathway identified by participants was the role of media, which emerged as a complex factor. The dual role of media, as both a potential contributor to delusional content among individuals with psychotic disorders and as a platform for raising mental health awareness more broadly, underscores that media exposure can carry both protective and risk‐related implications for mental health. A participant cautioned against alarmist messaging that may inadvertently heighten distress without offering constructive pathways for action. This tension is well recognized in the eco‐anxiety literature, which describes eco‐anxiety as a chronic fear of environmental doom that can manifest as persistent worry, helplessness, and distress in response to climate‐related threats [18]. While raising public awareness about climate change is important, messaging that emphasizes only the negative outcomes without offering meaningful individual or collective actions can deepen psychological distress [18]. Therefore, climate change communication should combine awareness with solution‐orientated messaging and clear calls for action, rather than avoiding discussion of severe outcomes altogether.

Lastly, participants consistently called for a system‐oriented and transdisciplinary approach, arguing that siloed approaches across disciplines and government departments are insufficient for addressing the interconnected nature of climate‐related mental health challenges. This call resonates with planetary health frameworks that advocate for coordinated action across public health, environmental science, clinical practice, and policy to develop effective and contextually‐relevant interventions [28]. In practice, such an approach could involve collaboration between mental health professionals, public health practitioners, environmental scientists, policymakers, community organisations, and civil society groups. For example, climate and weather surveillance systems could be integrated with public health and mental health monitoring systems to identify communities at increased risk following extreme weather events. At the community level, partnerships between local government, healthcare services, schools, and non‐governmental organisations could support mental health promotion, psychosocial support, and climate adaptation initiatives targeted towards vulnerable populations. Such coordinated efforts may help ensure that responses address both the environmental drivers of climate change and the social determinants that shape mental health vulnerability.

A major strength of this study was the use of purposive sampling, which ensured that participants were selected based on their relevant expertise and experience. The inclusion of active researchers, research funders, and ethics committee members is a particular strength, as these perspectives are often neglected in the existing literature on climate change and mental health. However, several limitations should be acknowledged. Although a sample of twelve participants is on the lower end for qualitative inquiry, recruitment was stopped upon achieving data saturation, meaning that no new themes emerged toward the end of the data collection process, which provides confidence in the sufficiency of the data. An *n* = 12 sample is well‐established as methodologically rigorous for achieving thematic saturation in highly specialised, elite‐informant qualitative studies where participants share deeply specific institutional knowledge. The recruitment of employees of the organization that funds climate change and/or health research, in this case all government employees, was limited by their seniority, specialized roles, and restricted institutional access, a known challenge in qualitative health policy research. The perspectives captured here, while not exhaustive, represent a rare contribution from this hard‐to‐reach stakeholder group, and findings should be interpreted accordingly as reflecting the participants interviewed rather than the full range of stakeholder views. As this study intentionally focused on capturing specific expert perspectives, the voices or expertise of people with lived mental health conditions were not included. Furthermore, although participants were selected on the basis of professional expertise, it cannot be ruled out that their responses were influenced by their own personal experiences of climate change or mental health, which may have shaped their perspectives in ways not accounted for in the analysis. Future research would benefit from complementing these expert insights with the lived experiences of individuals directly affected by climate‐related mental health challenges.

Future research should also seek to engage larger and more diverse samples, including community members, non‐governmental organizations, community‐based practitioners, and those with lived experience, to deepen and validate these findings across different contexts within South Africa and beyond.

## 4 Conclusion

This study contributes to a growing body of qualitative evidence that climate change poses multifaceted risks to mental health while drawing attention to the distinctive resilience that exists within affected communities, offering a more nuanced understanding of how individuals and communities in South Africa navigate the psychological consequences of a changing climate.

The findings underscore that in South Africa, where poverty, food insecurity, and limited healthcare resources remain pervasive, the psychological consequences of climate change are inseparable from the structural inequalities that continue to define the everyday lives of the most vulnerable communities. The rich expert perspectives captured in this study point toward a promising path forward, one in which researchers, clinicians, funders, and policymakers work collaboratively across sectors to develop integrated and contextually grounded responses that are both scientifically rigorous and deeply attuned to the lived realities of communities most affected by climate change in South Africa.

## Author Contributions

**Andrew Tomita:** conceptualization, writing – original draft, writing – review and editing, methodology, funding acquisition, project administration, validation, supervision. **Princess Nyoni:** writing – original draft, writing – review and editing, formal analysis, data curation, methodology, validation. **Joyce Mlay:** writing – original draft, writing – review and editing, methodology, formal analysis, data curation, validation. **Jessica Garlick:** writing – original draft, writing – review and editing. **Rosebub Gugulethu Gcaba:** writing – original draft, writing – review and editing, data curation. **Thobile Maphumulo:** writing – original draft, writing – review and editing, data curation. **Helga Chauke:** writing – original draft, writing – review and editing, data curation, project administration, methodology, validation, supervision. **Richard Lessells:** conceptualization, writing – original draft, writing – review and editing, methodology, validation, supervision. **Admire Nyamwanza:** conceptualization, funding acquisition, writing – original draft, writing – review and editing, methodology, validation, project administration, supervision.

## Ethics Statement

Written informed consent was obtained from all participants prior to participation. The study was approved by the Biomedical Research Ethics Committee at University of KwaZulu‐Natal (BREC/00007907/2024). Our study adhered to the Declaration of Helsinki.

## Conflicts of Interest

The authors declare no conflicts of interest.

## Transparency Statement

The lead author (manuscript guarantor) affirms that this manuscript is an honest, accurate, and transparent account of the study being reported; that no important aspects of the study have been omitted; and that any discrepancies from the study as planned have been explained.

## Data Availability

The data that support the findings of this study are available on request from the corresponding author. The data are not publicly available due to privacy or ethical restrictions.
